# FIP 1.0 soybean data: Insights on soybean growth from eight years of high-throughput image field phenotyping

**DOI:** 10.1038/s41597-026-06663-z

**Published:** 2026-02-18

**Authors:** Beat Keller, Norbert Kirchgessner, Corina Oppliger, Lukas Kronenberg, Lukas Roth, Olivia Zumsteg, Simon Corrado, Frank Liebisch, Helge Aasen, Nicola Storni, Flavian Tschurr, Hansueli Zellweger, Claude-Alain Betrix, Christoph Barendregt, Andreas Hund, Achim Walter

**Affiliations:** 1 Crop Science, Institute of Agricultural Science, ETH Zürich, 8048 Zurich, Switzerland; 2 Plant Breeding, Agroscope, 1260 Nyon, Switzerland; 3 DSP Delley Seeds, 1567 Delley, Switzerland; 4 Present Address: Waterprotection and substance flows, Agroscope, 8046 Zürich, Switzerland

**Keywords:** Plant breeding, Agroecology

## Abstract

Soybean growth is determined by the interaction of genetic, environmental, and management factors. In the context of future climate and climate extremes, understanding genotype by environment interaction (GxE) will be crucial for selecting resilient breeding lines and optimizing management practices to minimize stress. This requires an in depth elucidation of stressful weather conditions and differing temporal responses of genotypes to those conditions. In field studies, however, the environment is often treated as a static factor, and the specific effects of weather variability on crop growth remain poorly understood. Here, we present a longitudinal dataset comprising 17,247 high-resolution RGB images of soybean breeding lines collected throughout eight years in Eschikon, Switzerland. Top-of-canopy images were acquired throughout the entire growing seasons and complemented by hourly weather data, enabling a comprehensive analysis of soybean growth dynamics under varying field conditions. High spatio-temporal image resolution allows detailed analysis of growth dynamics and GxE, supporting identification of stress-tolerant genotypes to improve yield prediction and yield stability.

## Background & Summary

Understanding genotype by environment interaction (GxE) under field conditions is essential for improving crop resilience and yield stability. While distinguishing genotypic effects between different environments is valuable, a more detailed understanding of how weather variables affect the growth of specific genotypes is crucial. Among the weather variables, radiation and water availability play a dominant role in determining photosynthetic efficiency, while temperature influences physiological reactions and enzymatic function^1^.

Crop growth models (CGM) such as CROPGRO^2,3^ and APSIM^4,5^ have been widely used to predict crop performance under different environmental scenarios^6^. Kothari *et al*.^7^ suggest that improving model accuracy requires better input data, particularly for leaf expansion and senescence dynamics^7^. Canopy cover (CC) is often used as a proxy for plant growth and biomass accumulation, and it is commonly assessed using RGB images captured by Unmanned Aerial Vehicles (UAV)^8,9^, while digital repeat photography (“Phenocams”) have been used to track crop development and phenological indicators^10^. However, imaging approaches with high spatio-temporal resolution remain underutilized.

High Throughput Field Phenotyping (HTFP) enables a rapid and frequent collection of large-scale phenotypic data, which is crucial for: *i)* Identifying promising genotypes based on growth traits (including organ segmentation and counting, biomass estimation) that correlate with yield and stress resilience. *ii)* Capturing GxE to specific weather conditions, including extreme temperatures, drought, and varying light intensities. *iii)* Providing high-resolution temporal data to refine CGMs and improve their predictive capabilities for different environments. *iv)* Facilitating decision-making for crop/weeed identification or optimizing management practices, such as irrigation and fertilization, based on actual crop conditions^11–13^.

In this data collection, we used a stationary, rope-suspended camera system that autonomously captures top-of-canopy images at high spatial and temporal resolution. This Field Imaging Platform (FIP) enables the precise monitoring of plant growth at the plot level in short time intervals at the platform’s location^14^. By integrating high-resolution RGB imaging with meteorological data over several years, this data set aims to bridge the gap between genotype and environment (and management, although management and year are confounded in this dataset), enabling valuable insights into soybean growth dynamics.

The benefit of such high-resolution image data was demonstrated by^15^ incorporating cultivar-specific models that account for per se temperature responses in a combined soybean and wheat data set including a subset of images (two genotypes, three years) presented in this study. Such detailed phenotypic information is instrumental in refining thermal time concepts and improving the accuracy of genotype-specific predictions in CGM under varying climatic conditions. In wheat, a FIP derived wheat data set was recently made publicly available^16^. Furthermore, images from that data set were used to train a plant segmentation model^17^. In peas, individual flowering detection was successfully performed with high accuracy using FIP images^18^.

These advancements are relevant for growth prediction using CGM and improve selection efficiency for new soybean varieties. Such varieties are needed in European agriculture transitioning into more resilient and sustainable agricultural systems.

## Methods

### Plant material

Soybean breeding lines and varieties were obtained from Agroscope Soybean Breeding and Delley seeds and plants Ltd. (DSP) for performance evaluation from 2015 to 2022. In total, 72 soybean genotypes were evaluated including 58 breeding lines, 14 varieties as checks and three mixtures of two varieties. The lines included maturity types ranging from the very early (000) type to the intermediate (II) type^19^ and most were bred by Agroscope/DSP (Supplementary Table 1).

### Field trials

Field trials were carried out at ETH research station of plant sciences in Lindau Eschikon, Switzerland (47.449 N, 8.682 E, 556 m a.s.l.). The soil type is an eutric cambisol with an organic matter content of around 3.5% and a pH between 6.2 and 7. Soybean was following winter wheat and green manure in crop rotation.

Common treatments across all years included soil preparation with plowing and harrowing, herbicide applications for weed control, and fertilization based on soil test results. Detailed treatment, dates of sowing and harvest of each trial are shown in Supplementary Table 2. Sowing density varied between 40 and 60 plants m^−^^2^.

From 2015 to 2022, eight trials (one trial per year) were carried out, including 11 to 36 genotypes in a complete randomized block design. In 2015, eight replicates were used. From 2016 to 2017, three replicates and one to six check varieties with six to twelve replicates were used. The trials from 2018 to 2020 contained three replicates and were described before^20^.

### Yield and reference traits

The harvest was processed as described in Roth *et al*.^20^. Moisture and protein content was measured using near-infrared spectroscopy (NIRS) (A 7200 NIR Diode Array Based Analyzer, Perten Instruments NA, Inc., Springfield, USA) on an approximately 100 g subsample of the harvested grain per plot. In 2018 and 2022, both traits were measured in all three replicates. In the remaining years, only one mixed sample of the three replicates was measured. The weighed seed yield and protein content were corrected for moisture content of 11% based on the available values. Handheld chlorophyll meter measurement (SPAD) were taken manually.

Plant heights were measured using a Focus3D S 120 terrestrial laser scanner (TLS) (Faro Technologies Inc., Lake Mary USA) mounted on the FIP in 2016 and 2017. Height was extracted as the 97^th^ percentile of the resulting height profile^21^.

### Image acquisition

**Fig. 1:**
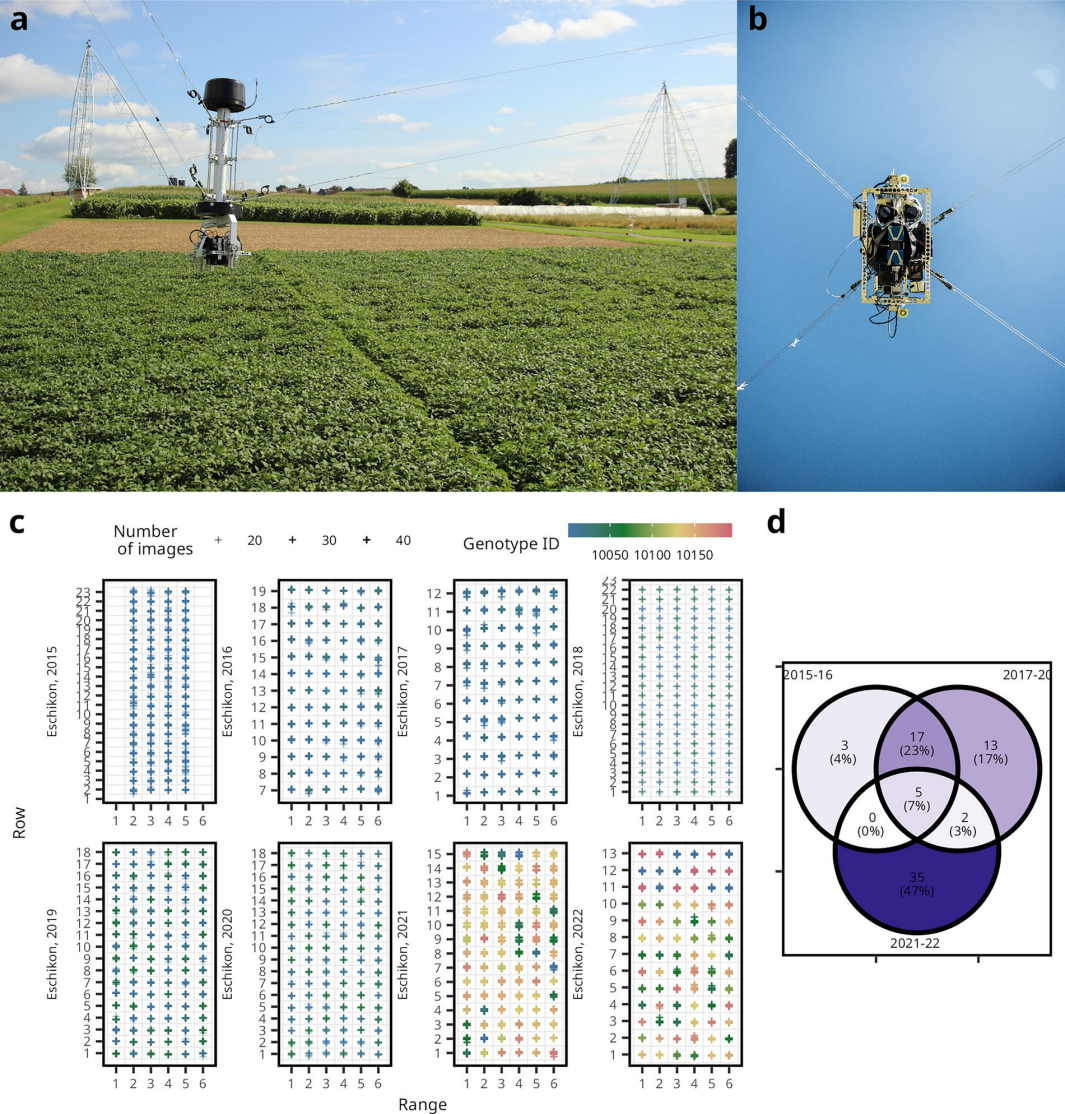
Autonomous monitoring of soybean field trials using the rope-suspended Field Imaging Platform (FIP). (**a**) The FIP is operating over a soybean field trial. (**b**) The FIP camera head in a bottom-up view reveals the camera lens. (**c**) The experimental plots of all eight trials are arranged in their respective rows and colums. The cross symbols represent the estimated image acquisition positions. The colors indicate the genotypes and the transparency indicates the number of measuring dates, i.e., the number of images per plot. (**d**) In total, 72 genotypes were tested with various overlap between the years.

The images were acquired using the FIP, a stationary, rope-suspended imaging platform (Fig. 1). The images were taken as described in Kirchgessner *et al*. (2017) from nadir view using an EOS 5D Mark II with a 35 mm lens mounted (Canon Inc., Tokyo, Japan)^14^. The camera was positioned 3 m above the canopy, the images were taken at full resolution of 21 MP in raw mode with usually 4 ms exposure time (Supplementary Table 3). The ground sampling distance was approximately 0.55 mm. Additional camera parameters and file name structure are described in Supplementary Table 4.

### Image analysis and plot detection

**Fig. 2:**
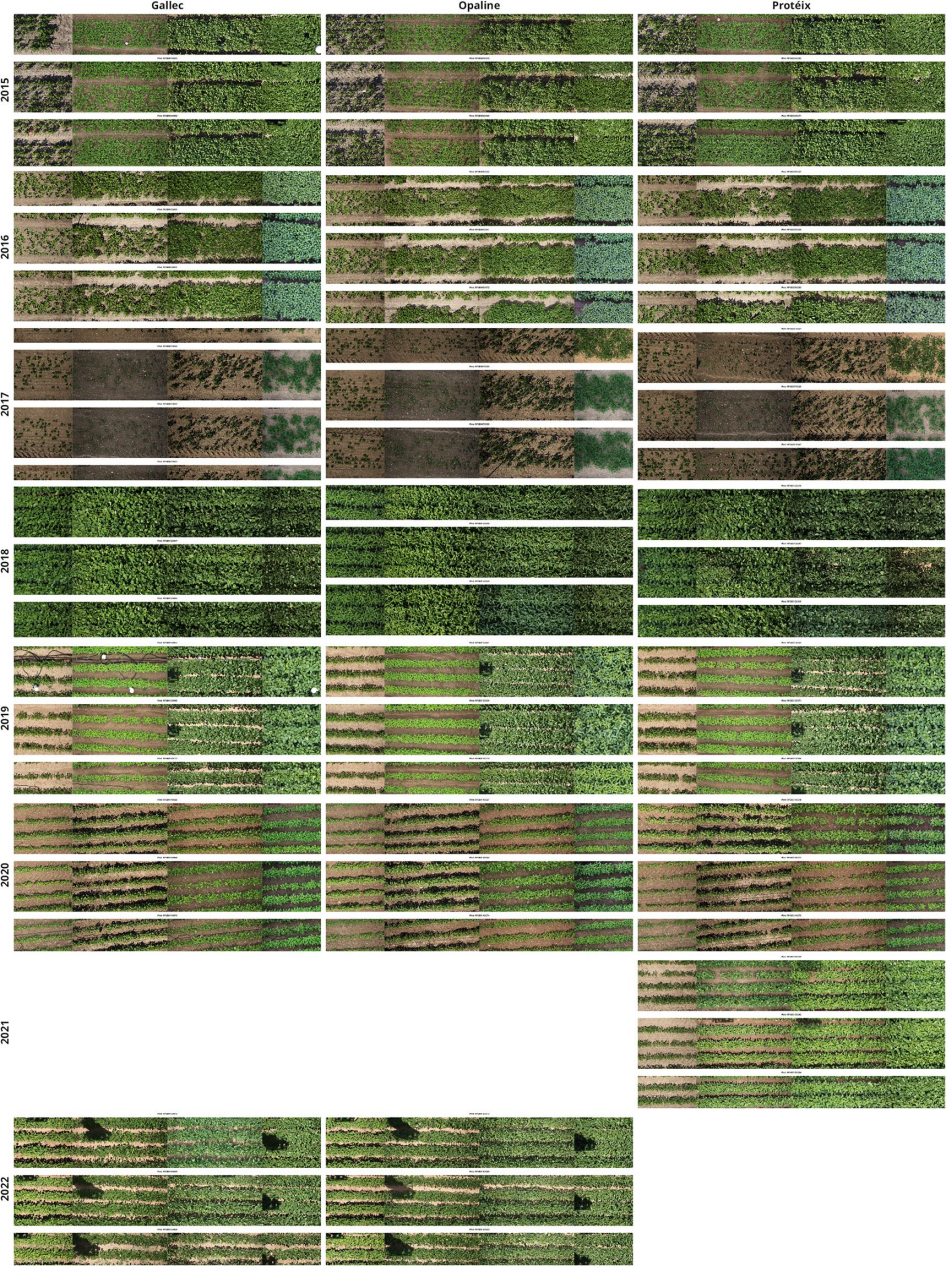
Representative top-of-canopy RGB images of three exemplary soybean genotypes captured by the Field Imaging Platform (FIP) over eight years. The selection includes the lanceolate leaf type variety *Opaline*. Four consecutive image dates illustrate distinct morphological growth stages across different years, demonstrating the temporal resolution and image quality of the dataset.

**Fig. 3:**
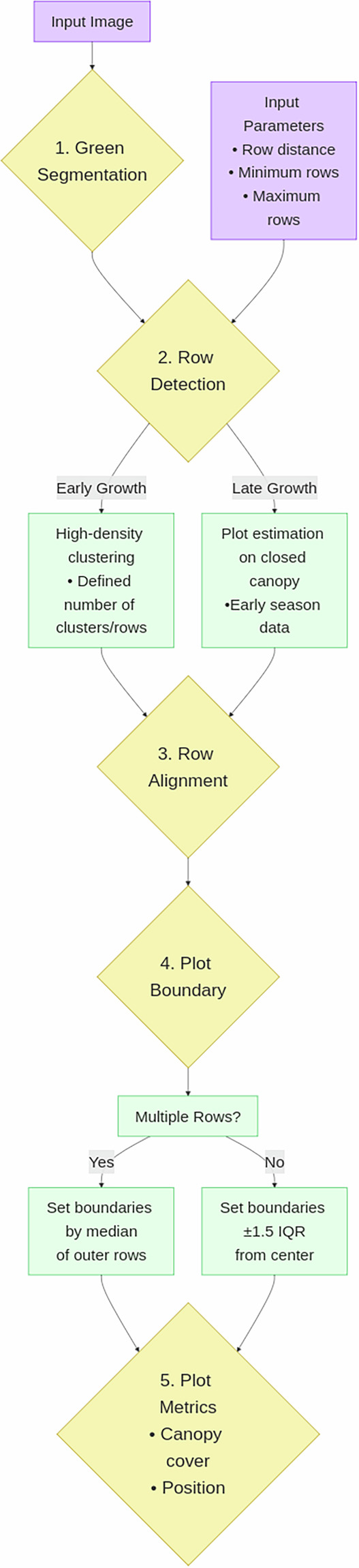
The flowchart illustrates the process steps for green canopy cover (CC) extraction from RGB plot images. Violet nodes indicate the input canopy images and required parameters, yellow nodes represent the main processing steps, and green nodes provide explanations of the included processes.

**Fig. 4:**
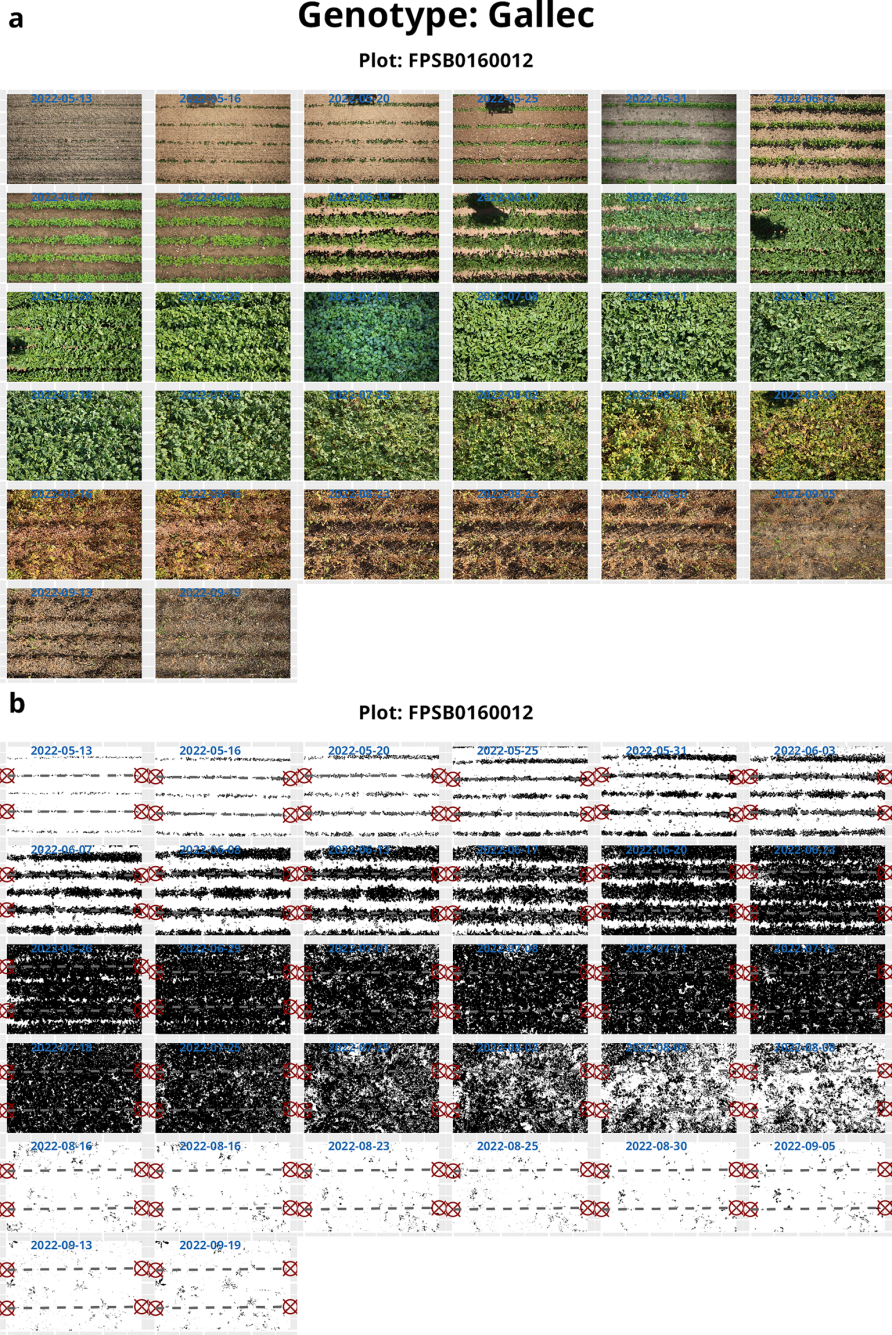
Example of plot detection and green canopy cover (CC) extraction for the variety *Gallec* based on images acquired throughout the season using the Field Imaging Platform (FIP). (**a**) Original RGB images were taken at different growth stages throughout the 2022 season. (**b**) Plot detection for extracting green CC from segmented images is demonstrated. Plot boundaries were defined between the three central rows per image. During later growth stages, when the canopy closed and individual rows or plots could no longer be distinguished, plot boundaries were inferred from earlier-season images.

Without geo-referencing of the images, the identification of plots has two main obstacles in images taken over time: The plot location in the image is unknown, and the (unknown) plot position varies due to variation in the camera position (Fig. 2). To resolve these issues, we developed an algorithm which uses the approximate row distance, the minimum rows (in the plot) and maximum rows (of the image) as parameters (Fig. 3). The following five steps were taken to extract plot-based green CC values:

1. A green-pixel segmentation was performed using color ratio thresholds based on an established leaf area segmentation algorithm^22^. Segmented images are saved as compressed jpg images at 90% quality for potential further use by other users.
2. When plant rows were still visible before canopy closure, clusters were identified using only vertical (y-axis) pixel distribution. This approach grouped pixels into horizontal bands corresponding to plant rows using high-density clustering, effectively ignoring gaps along the x-axis caused by incomplete emergence (Supplementary Fig. 1). This resulted in a maximum of five and a minimum of three predefined clusters, i.e., rows, in a three-row plot. The cluster nearest to the image center was identified as the middle row, and neighboring clusters were numbered based on their proximity. Excess rows were dropped if they exceeded the defined cluster maximum and if they spanned over two or more rows, according to the pre-defined row spacing. This ensured stable row extraction when, e.g., weeds were present within the rows.
3. Rows were then rotated around their midpoint aligning them horizontally and parallel to each other, thereby correcting for both perspective distortion and camera misalignment relative to the plot axis, using robust linear regression.
4. Plot-level green CC was extracted together with pixel counts and positional metadata of the identified plot area (Supplementary Table 5). The CC was calculated by determining the proportion of green pixels relativ to the total number of pixels in the plot area (Supplementary Table 6). If more than one row was detected, the upper and lower plot boundary was defined by the median value of the outer rows. If just one row was present (this was used for the nine row plots as they are hard to separate), the upper and lower plot boundaries were set to 1.5 interquantile ranges off-center. For later stages of plant growth, when the canopy was closed and individual rows and/or plots could not be clearly distinguished, plot boundaries were estimated from images earlier in the season.
5. Finally, the two complementary datasets—one based on total green canopy fraction and a row-based data set were merged to get the canopy dynamics over the season (for the resulting csv file see: data/Soybean_CanopyCover_Raw_data.csv). An example of the workflow including plot localizing and CC extraction in a single plot over time is shown in Fig. 4.

### Data Analysis For Technical Validation

Best linear unbiased estimators (BLUEs) for each trait and trial were estimated using the Spatial Analysis of Field Trials with Splines (SpATS) R package^23^ as the following:

$${y}_{ijk}=\mu +{{\rm{gen}}}_{i}+{f}_{u,v}({{\rm{col}}}_{j},{{\rm{row}}}_{k})+{\varepsilon }_{ijk}$$

where *y*_*ijk*_ denotes the observed phenotypic plot value for genotype *i* at column *j* and row *k*, *μ* is the overall mean, gen_*i*_ is a fixed effect of genotype *i*, *f*_*u*,*v*_(col_*j*_, row_*k*_) models spatial variation using bilinear polynomial and smoothing spline functions over the column and row positions, and *ε*_*ijk*_ is the residual error term. A plot value was removed from the analysis when the residuals of the fitted plot value were larger than  ±3 times the residual standard deviation as described previously^24,25^. For heritability calculation, genotype was set as random factor.

## Data Records

The FIP 1.0 soybean dataset is available in the ETH Zurich Research Collection (10.3929/ethz-b-000742401)^26^, and is also provided as a Hugging Face dataset card (10.57967/hf/6052)^27^ allowing interoperability and standardization with other datasets. In the presented dataset, the FIP was successfully deployed to phenotype a wide range of soybean genotypes over entire field seasons (Fig. 1a). Top-of-canopy imaging was conducted using high-resolution RGB cameras mounted approximately 3 m above the plots (Fig. 1b).

Across the eight field trials conducted between 2015 and 2022, a total of 17,247 canopy images were collected, of which 1,098 images were excluded from the subsequent analysis because they showed border plots. The data set represents 72 soybean genotypes (Fig. 1c). The number of genotypes per year ranged from 11 to 36, including five standard check varieties that were consistently grown across the trials (Fig. 1d). Management varied slightly between the years, e.g., from 2015 to 2018 nine planting rows per plot were sown, afterwards plots were sown in three rows (Supplementary Table 2). The associated yield, height and reference trait data is additionally provided.

Weather data was provided by the Swiss Federal Agrometeo (https://agrometeo.ch/) station Lindau (47.446 N, 8.680 E, 551 m a.s.l.) at hourly resolution. At this station the radiation sensor failed for one season, therefore, from 2020 to 2022, photosynthetic photon fluence rate (PPFR) was taken from a LI-COR sensor adjacent to the FIP field and aggregated to hourly resolution. Correlations between PPFR values from both sensors were assessed using overlapping hourly observations to confirm measurement consistency (Supplementary Fig. 2). The factor to convert radiation in MJ m^−^^2^ to PPFR was 2.04 according to^28^.

### Data Files and Structure

The dataset is structured into directories that align with the described data processing pipeline used for extracting and analyzing CC traits from field images. All files are provided in interoperable and widely-used ‘.csv` and ‘.png` format.

**Fig. 5:**
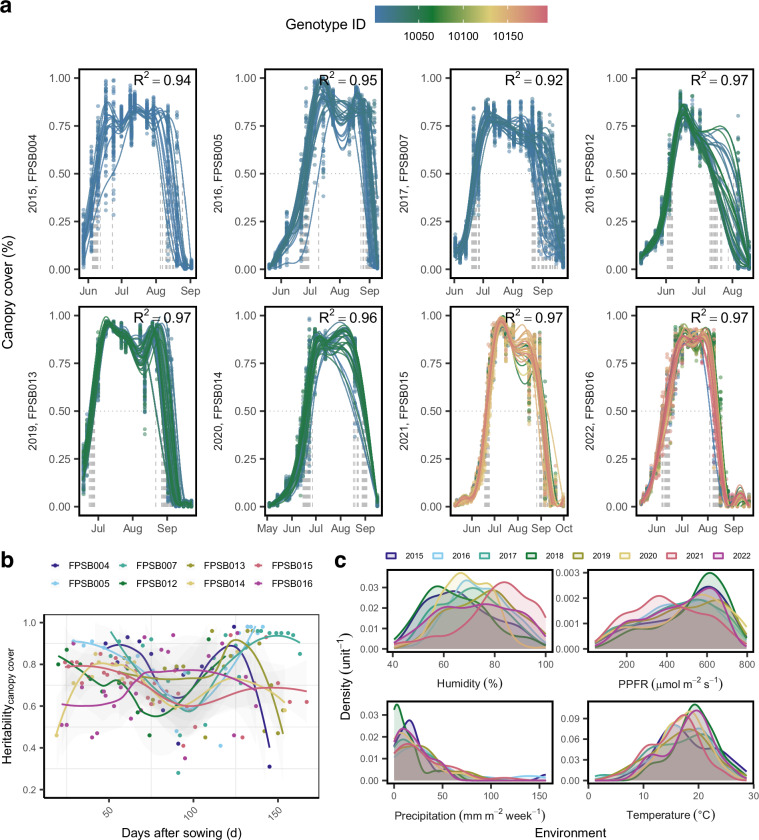
The green canopy cover (CC) of soybean genotypes including elite breeding lines and check varieties over eight years. (**a**) The raise and decline of CC is fitted by a generalized additive model (GAM) for each genotype. Vertical dotted lines indicate that 50% CC are reached. (**b**) Heritabilities from CC values for each date were derived based on Spatial Analysis of Field Trials with Splines (SpATS). (**c**) Kernel density plots show the distribution of weather variables during the growing season.
- data/Soybean_CanopyCover_BLUEs.csv: Contains BLUEs of CC for each genotype, date and environment, derived from spatial modeling using the SpATS package^23^. Each data record includes adjusted means, standard errors, and heritability estimates (Fig. 5b), providing genotype-level summaries for subsequent analysis and overall data quality control (Fig. 5b).
- data/Weather_data_Lindau_Eschikon.csv: Provides hourly weather records (temperature, radiation, PPFR, humidity, precipitation, windspeed) collected at the experimental site. The variables can be aligned with image acquisition dates and data (Fig. 5c and Supplementary Fig. 3) to investigate weather effects on CC development.
- data/RefTraits_Soybean_Eschikon_2015_22_BLUEs.csv: The BLUEs for grain yield, protein content, protein yield, Thousand Kernel Weight (TKW) and SPAD were calculated for each available genotype and trial. This file enables integration of CC data with agronomic traits (Fig. 6).

The following files are included in the dataset, organized according to the processing workflow and their intended analytical purpose:

- data/Design_2015_2022_Eschikon.csv: Experimental design file, including plot layout (row and range), genotype names and unique plot identifiers (plot UID), and replication. This file links the image-based data to the respective field trials and is essential for adding experimental meta data as genotype name to plot data.
- raw/: Contains original nadir (top-view) images acquired from the FIP saved as raw CR2 (Fig. 2). Each image is uniquely named, encoding the date, field trial, and the plot UID (Supplementary Table 4).
- segmentation/: Includes binary segmentation masks generated through green filtering, stored as ‘.png` images. These masks differentiate green canopy from the soil background, and are used for downstream plot-based CC extraction (Fig. 4).
- data/Soybean_CanopyCover_Raw_data.csv: Contains raw parameters extracted per segmented image for calculating CC, including the number and position of detected rows, per-plot row spacing estimates, plot edges coordinates, and extracted green pixel counts within the identified plot area. This data can be used to refine the CC extraction procedure.
- data/Soybean_CanopyCover_data.csv: Provides cleaned and normalized CC values, with each observation tied to the plot UID, date, time, and genotype. The CC was robustly extracted for each plot and corrected by the determined row width (Fig. 5a). This dataset can be directly used for CC modeling and subsequent analyses at plot-level.

**Fig. 6:**
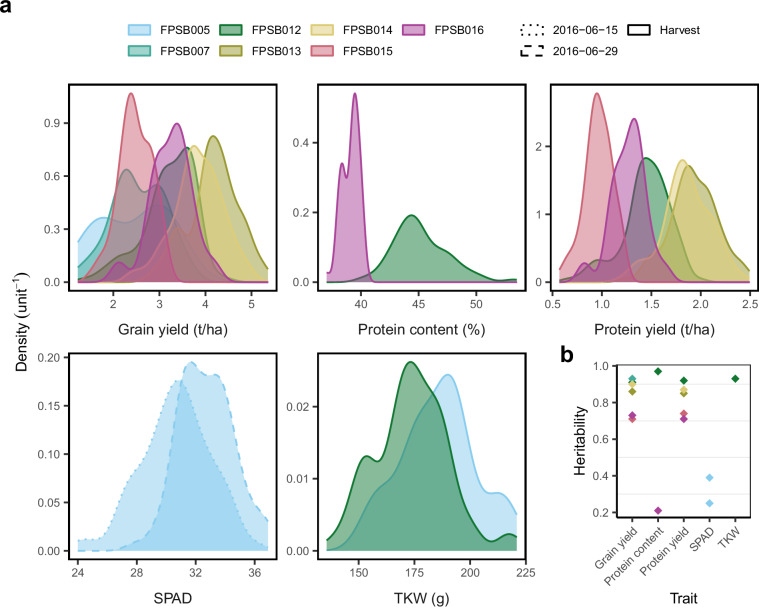
Reference traits of soybean genotypes across multi-year trials. (**a**) Kernel density plots show the distributions of agronomic traits for the soybean genotypes in each trial. (**b**) Broad-sense heritabilities of the traits were derived based on Spatial Analysis of Field Trials with Splines (SpATS).

To ensure interoperability, variable names follow MIAPPE^29^ naming conventions were included (Supplementary Table 7). Further harmonization with other public datasets may be achieved using the metadata fields for genotype names and trial dates, e.g., accessing our data via Hugging Face (10.57967/hf/6052).

## Technical Validation

Several validation steps were performed to ensure high quality of the dataset. First, designs were visually checked for the correct position of the genotypes based on the images, especially the lancelote leaf type *Opaline* (Fig. 2). Segmentation performance using green filtering was visually confirmed by overlaying extracted green canopy areas on the original images of at least one plot over the season (Supplementary Fig. 4). In addition, heritability of extracted CC was assessed. Broad-sense heritability of CC was estimated for each time point, with values ranging from 0 to 0.98 depending on growth stage and field conditions. Dates with heritability values lower than 0.2 were then removed as they likely do not add genotypic information (these were six measuring dates in the latest senescence phase). On the remaining data, a high median heritability value of 0.76 ± 0.16 supports the robustness of the phenotyping process (Fig. 5b). The reference traits reached heritability values ranging from 0.71 to 0.93 for yield and from 0.21 to 0.97 for protein content.

Curves were fitted for CC using generalized additive model (GAM) to assess smoothed temporal trends over time^30^. The model was fitted for each genotype separately in each trial using:

1 $${y}_{i}={\beta }_{0}+f({x}_{i})+{\varepsilon }_{i}$$

where *y*_*i*_ is the green CC measurement at observation (date and plot) *i*, *β*_0_ is the intercept term, *f*(*x*_*i*_) is a smoothing function of the predictor, i.e., the numeric date *x*_*i*_, and ${\varepsilon }_{i} \sim {\mathcal{N}}(0,{\sigma }^{2})$ is the normally distributed error term. The smoothing function *f*(*x*) is modeled using penalized regression splines. The number of basis functions used to construct each smoothing term was set to ten (k=10).

The coefficient of determination (*R*^2^) for the smoothed curve was calculated for each field site and year combination, with all *R*^2^ values above 0.92, indicating high temporal coherence of the data (Fig. 5a).

**Fig. 7:**
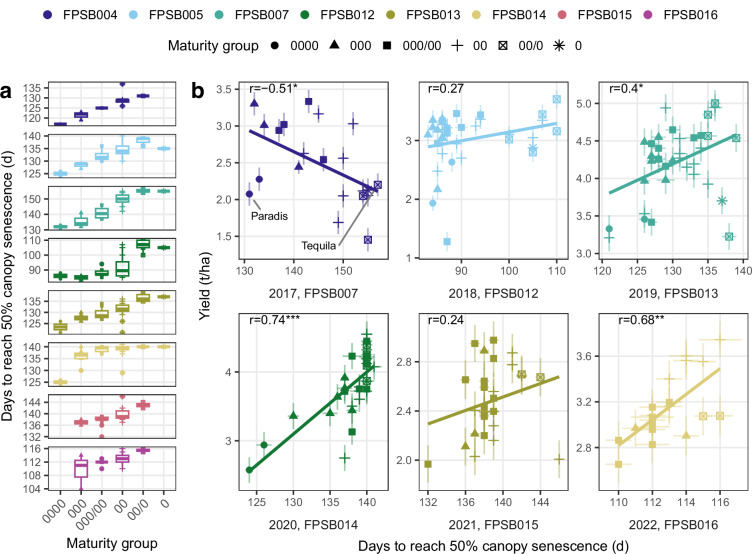
Relationship between senescence timing (days to reach 50% canopy senescence) maturity group and yield across soybean trials. Maturity groups range from very early (0000) to early (0). (**a**) Boxplots represent senescence timing across maturity groups within each trial. Boxes represent the interquartile range (IQR; 25th to 75th percentile), horizontal lines indicate the median, and whiskers extend to 1.5  × IQR. Senescence timing was estimated from fitted CC for each genotype and trial using generalized additive model (GAM). (**b**) Correlation between senescence timing and grain yield are presented for each environment. Pearson’s correlation coefficients (r) are shown with significance levels for *p*-values indicated by *p < 0.05, **p < 0.01, and ***p < 0.001. Each point represents adjusted genotypic values. Grey error bars show standard error. The senescence timing corresponds to the point where green CC has declined to 50% after canopy closure. Intermediate maturity groups (I and I/II) were excluded because their senescence was too delayed to extract this point. Extreme genotypes in senescence timing in 2017 (trial FPSB007) are labeled, and their image time series shown in Supplementary Fig. 5.

Additionally, we computed the date when each genotype reached 50% green CC by interpolating fitted curves before and after reaching canopy closure. These dates, representing the senescence timing, were consistent with reported maturity groups of the genotypes, revealing distinct senescence dynamics among them (Fig. 7a). Furthermore, the senescence timing correlated with yield, reaching Pearson’s correlation coefficient of up to 0.73 (Fig. 7b). The correlation was negative in the 2017 season (FPSB007), where late-maturity groups (00/0 and 00) exhibited delayed senescence without reaching their yield potential (Supplementary Fig. 5), contrary to the generally higher yields typically associated with later maturity types^31,32^.

**Fig. 8:**
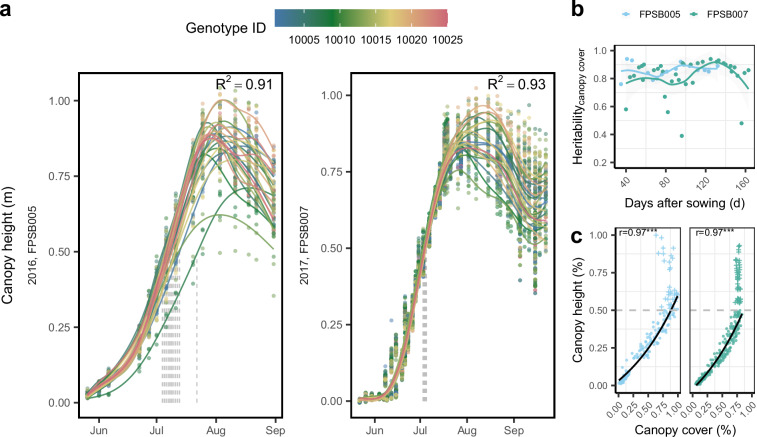
The canopy height of genotypes including elite breeding lines and check varieties over two years. (**a**) The raise and decline of canopy height is fitted by a generalized additive model (GAM) for each genotype. Vertical dotted lines indicate that 50% canopy height are reached. (**b**) Heritabilities from canopy height values for each date are shown. (**c**) Correlation between canopy height and canopy cover are presented for each environment until reaching 50% of canopy height. The threshold is indicated by the grey dashed line. Pearson’s correlation coefficients (r) are shown with significance levels for *p*-values indicated by *p < 0.05, **p < 0.01, ***p < 0.001. Each point represents adjusted genotypic value based on the GAM fit.

Similarly to CC, canopy height showed a logistic increase over time until reaching its maximum (Fig. 8a). The heritabilities over the two measured seasons were rather stable and high with a median value of 0.86 ± 0.22 (Fig. 8b). Until reaching 50% of its maximum, canopy height strongly correlated with extracted CC values reaching Pearson’s correlation coefficient of 0.97 (Fig. 8c).

## Usage Notes

The dataset can be readily used by researchers working on high-throughput phenotyping, crop modeling and deep learning (Supplementary Fig. 6).

Users interested in longitudinal canopy development can apply the included timestamp and plot UIDs to construct growth curves. For example, temporal fits using GAM or smoothing splines can reveal genotype-specific differences in growth dynamics. Including weather variables enables insights into GxE, for example, how the CC dynamics of genotypes differ in response to temperature variation. Since management practices varied slightly between years (e.g., sowing density and number of rows per plot), management and year effects are confounded. We therefore recommend treating *year* as a random effect in mixed-model or GxE analyses to account for this structure and to avoid overestimating management-related effects. The current dataset can be easily combined with other datasets, including such originating from UAV-based phenotyping, to enable broader insights into growth dynamics.

Besides green CC, more detailed information can be extracted from images such as organ segmentation and potentially pod count^33–35^.

## Supplementary information

Supplementary Information

## Data Availability

The code is available on: https://gitlab.ethz.ch/crop_phenotyping/fip-soybean-canopycover. Users with similar data can use the implemented workflow to get canopy cover from their experiments.
