# Supplementary material for: FIP 1.0 soybean data: Insights on soybean growth from eight years of high-throughput image field phenotyping
Source: Sci Data. 2026 Feb 18;13:476. doi: 10.1038/s41597-026-06663-z (PMC13031312; doi:10.1038/s41597-026-06663-z)
Supplement: Supplementary file 1 — Supplementary Information [file 41597_2026_6663_MOESM1_ESM.pdf]

Supplementary Information

Supplementary Tables

Detailed description of genotypes

Supplementary Table 1: Genotype Information

No.	Genotype ID	Genotype Name	Breeder	Registration	Maturity Group
1	10001	Gallec	Agroscope/DSP	CH:2000	000
2	10002	Lissabon	Saatbau Linz	AT:2012	00
3	10003	Protéix	Agroscope/DSP	CH:2009	00
4	10004	Merlin	Saatbau Linz	AT:2007	000
5	10005	Obelix	Agroscope/DSP	CH:2014	000
6	10006	Aveline	Agroscope/DSP	CH:2005	000/00
7	10007	Tourmaline	Agroscope/DSP	CH:2013	00
8	10008	Amphor	Rustica	FR:2001	00
9	10009	Opaline	Agroscope	CH:2009	00
10	10010	Tiguan	Agroscope/DSP	CH:2014	0000
11	10011	Falbala	Agroscope/DSP	CH:2013	00/0
12	10012	Paradis	Agroscope/DSP	CH:2000	0000
13	10013	Protibus	Agroscope/DSP	CH:2013	00
14	10014	Amarok	Agroscope/DSP	CH:2014	000/00
15	10015	Coraline	Agroscope/DSP	CH:2014	00
16	10016	Galice	Agroscope/DSP	CH:2015	000/00
17	10017	Toutatis	Agroscope/DSP	CH:2015	000/00
18	10018	Tequila	Agroscope/DSP	CH:2013	00/0
19	10019	Bagera	Agroscope/DSP	CH:2007	00/0
20	10020	Idefix	Agroscope/DSP	CH:2002	0
21	10021	Pollux	Agroscope/DSP	CH:2012	00
22	10022	Orion	Agroscope/DSP	NA	0
23	10023	Castétis	Agroscope/DSP	IT:2010	I
24	10024	Paco	Agroscope/DSP	IT:2012	I/II
25	10025	MapleArrow	a	CA:1976	00
26	10044	22460	Agroscope/DSP	NA	000
27	10045	Navaro	Agroscope/DSP	CH:2020	000
28	10046	Paprika	Agroscope/DSP	CH:2020	000/00
29	10047	22511	Agroscope/DSP	NA	00
30	10048	CH90008	Agroscope/DSP	NA	000
31	10049	Amandine	Agroscope/DSP	CH:2012	000
32	10050	Aurelina	SZD	NA	000/00
33	10051	Everest	Agroscope/DSP	UA:2018	000
34	10052	Kalinka	Agroscope/DSP	CH:2017	00
35	10053	Marquise	Agroscope/DSP	AT:2017	00
36	10054	Mentor	Lidea	NA	00/0
37	10055	Naskia	Agroscope/DSP	CH:2017	000/00
38	10056	Primus	Eurasia	NA	00
39	10057	Xena	Agroscope/DSP	CH:2017	000/00
40	10058	Yakari	Agroscope/DSP	F:2018	00/0
41	10065	Soramax	Agroscope/DSP	CH:2024	00
42	10067	Alambix	Agroscope/DSP	CH:2023	000/00
43	10073	CH90132	Agroscope/DSP	AT:2024	00
44	10074	CH90137	Agroscope/DSP	NA	00
45	10085	CH90139	Agroscope/DSP	NA	00

No.	Genotype ID	Genotype Name	Breeder	Registration	Maturity Group
46	10098	CH22778	Agroscope/DSP	NA	000/00
47	10100	CH22776	Agroscope/DSP	NA	00
48	10121	CH90082	Agroscope/DSP	NA	00
49	10122	CH22725	Agroscope/DSP	NA	00
50	10126	CH22713	Agroscope/DSP	NA	000/00
51	10128	CH22681	Agroscope/DSP	F:2024	00/0
52	10134	CH22707	Agroscope/DSP	CH:2024	000/00
53	10135	CH22706	Agroscope/DSP	NA	00
54	10136	CH22727	Agroscope/DSP	NA	000/00
55	10137	CH22715	Agroscope/DSP	NA	000/00
56	10139	CH22711	Agroscope/DSP	AT:2023	000/00
57	10140	CH22691	Agroscope/DSP	NA	00
58	10143	CH22738	Agroscope/DSP	NA	000
59	10144	CH22645	Agroscope/DSP	CH:2024	000/00
60	10145	CH22654	Agroscope/DSP	NA	000/00
61	10147	CH22751	Agroscope/DSP	NA	00
62	10148	CH22729	Agroscope/DSP	NA	000/00
63	10149	CH22650	Agroscope/DSP	NA	000/00
64	10152	CH22655	Agroscope/DSP	CH:2024	000/00
65	10155	CH22653	Agroscope/DSP	NA	000/00
66	10158	CH22730	Agroscope/DSP	NA	00
67	10163	CH90077	Agroscope/DSP	NA	00
68	10169	CH90075	Agroscope/DSP	CH:2023	00
69	10170	CH22624	Agroscope/DSP	CH:2023	000
70	10172	CH22517	Agroscope/DSP	CH:2024	000/00
71	10178	CH90126	Agroscope/DSP	NA	00
72	10179	Sirelia	Agroscope/DSP	NA	000
73	10192	Gallec.Opaline	NA	NA	NA
74	10193	Tourmaline.Opaline	NA	NA	NA
75	10194	Gallec.Tourmaline	NA	NA	NA

Detailed description of field trials

Supplementary Table 2: Summary of soybean field trials (2015–2022) in Eschikon, Switzerland (47.449 N, 8.682 E, 556 m a.s.l.).

Year	Task	Date	Treatment	Rate	Rows per plot
2015	Soil Preparation	2014-11-24	Plowing	-	-
	Fertilization	2015-03-11	Superphosphate, Foskal, Dolomit	150 kg/ha, 350 kg/ha, 400 kg/ha	-
	Sowing	2015-04-10	Seeding	60 plants/m²	9
	Weed Control	2015-04-10	Dual Gold, Molipan Pro	As recommended	-
	Weed Control	2015-04-14	Snail and slug bait	As recommended	-
	Weed Control	2015-05-28	Bolero	As recommended	-
	Weed Control	2015-06-10	Fusilade Max	As recommended	-
	Weed Control	2015-09-21	Roundup Power Max	As recommended	-
	Harvest	2015-09-08	Combine	-	-
2016	Soil Preparation	2016-03-15	Plowing	-	-
	Fertilization	2016-03-07	TSP 46, Kali	250 kg/ha, 350 kg/ha	-
	Sowing	2016-04-21	Seeding	-	9
	Weed Control	2016-04-21	Dual Gold, Molipan Pro	As recommended	-
	Weed Control	2016-04-21	Snail and slug bait	As recommended	-
	Weed Control	2016-06-01	Bolero	As recommended	-
	Weed Control	2016-06-01	Fusilade Max	As recommended	-
	Harvest	2016-09-28	Combine	-	-
2017	Soil Preparation	2017-02-27	Plowing	-	-
	Fertilization	2017-02-23	TSP 46, Kali	300 kg/ha, 400 kg/ha	-
	Sowing	2017-04-12	Seeding	40 plants/m²	9
	Weed Control	2017-04-13	Dual Gold, Molipan Pro	As recommended	-
	Weed Control	2017-04-18	Snail and slug bait	As recommended	-
	Weed Control	2017-05-19	Snail and slug bait	As recommended	-
	Weed Control	2017-06-01	Bolero	As recommended	-
	Weed Control	2017-06-01	Fusilade Max	As recommended	-
	Weed Control	2017-06-10	Bolero (repeat)	As recommended	-
	Harvest	2017-09-27	Combine	-	-
2018	Soil Preparation	2018-02-09	Plowing	-	-
	Fertilization	2018-02-06	TSP 46, Kali	300 kg/ha, 350 kg/ha	-
	Sowing	2018-04-19	Seeding	55 plants/m²	3
	Weed Control	2018-04-24	Dual Gold, Molipan Pro	As recommended	-
	Weed Control	2018-04-30	Snail and slug bait	As recommended	-
	Weed Control	2018-05-22	Fusilade Max	As recommended	-
	Weed Control	2018-06-01	Bolero	As recommended	-
	Harvest	2018-08-28	Combine	-	-
2019	Soil Preparation	2019-01-21	Plowing	-	-
	Fertilization	2019-02-27	TSP 46, Kali, Mg-Ammonsalpeter	200 kg/ha, 200 kg/ha, 225 kg/ha	-
	Sowing	2019-04-23	Seeding	55 plants/m²	3

(continued on next page)

(continued from previous page)

**Year**	**Treatment Stage**	**Date**	**Treatment**	**Rate**	**Rows per Plot**
	Weed Control	2019-04-24	Dual Gold, Molipan Pro	As recommended	-
	Harvest	2019-09-24	Combine	-	-
2020	Soil Preparation	2020-01-08	Plowing	-	-
	Fertilization	2020-03-13	TSP 46, Kali, Granumag	200 kg/ha, 200 kg/ha, 200 kg/ha	-
	Sowing	2020-04-15	Seeding	60 plants/m^2	3
	Weed Control	2020-04-16	Cargon S, Successor 600	As recommended	-
	Harvest	2020-09-21	Combine	-	-
2021	Soil Preparation	2021-01-05	Plowing	-	-
	Fertilization	2021-04-09	TSP 46, Kali, Granumag	200 kg/ha, 200 kg/ha, 200 kg/ha	-
	Sowing	2021-04-16	Seeding	60 plants/m^2	3
	Weed Control	2021-04-19	Cargon S, Successor 600	As recommended	-
	Weed Control	2021-06-02	Bolero	As recommended	-
	Harvest	2021-10-01	Combine	-	-
2022	Soil Preparation	2022-02-12	Plowing	-	-
	Fertilization	2022-03-08	TSP 46, Kali, Granumag	100 kg/ha, 150 kg/ha, 200 kg/ha	-
	Sowing	2022-04-21	Seeding	60 plants/m^2	3
	Weed Control	2022-04-22	Successor 600, Cargon S	As recommended	-
	Weed Control	2022-05-30	Bolero	As recommended	-
	Weed Control	2022-06-10	Bolero (repeat)	As recommended	-
	Weed Control	2022-06-10	Fusilade Max	As recommended	-
	Harvest	2022-09-23	Combine	-	-

Detailed camera acquisition description

Supplementary Table 3: Camera parameters per year, constant settings for all years: ISO = 100, White Balance = Auto white balance, Focal Length = 35 mm, Exposure Mode = Auto exposure, for all other parameters median values are shown.

**Year**	**F number**	**Aperture Value**	**Exposure bias value**	**Exposure program**	**Exposure time**
2018	16	8	-1	Aperture priority	0.016667
2019	4	4	0	Shutter priority	0.004
2020	8	6	-0.5	Shutter priority	0.004
2021	5.6	5	0	Shutter priority	0.004
2022	6.7	5.5	0	Shutter priority	0.004
all years	6.7	5.5	0	Shutter priority	0.004

Supplementary Table 4: Structure of the filenames and the information it contains

**Filename structure**	**Example**
Raw file name	FPSB0130031_RGB1_20190618_150650.CR2
Experiment name	FPSB013
Plot number	0031
Sensor name	RGB1
Date	20190618
Time	150650
File extension	CR2

Detailed description of canopy cover extraction

Segmentation

Green filter to extract canopy cover was applied as described in [1]. The pixel_check function analyzes an RGB image to segment green canopy from the background. It loads the images and iterates over each pixel, applying thresholding conditions to classify pixels as leaf, scale, or background based on minimum red (R) and green (G) values as well as ratios between red, green and blue. Identified green pixels are saved as a segmentation image, while the original image is resized and saved with 90% JPEG compression for further processing.

Plot detection without canopy closure

The function *get_row_pixels* processes the segmented images to extract and analyze plant rows based on pixel data. It trims unnecessary parts at the top and bottom based on *cut_above* and *cut_below* input values. It requires information about the maximum rows (*max_cluster*) expected to be visible in one image, the minimum rows (*min_cluster*) which define the rows belonging to the plot, and the expected row distance (*row_distance_min*) in pixels to ensure consistent row detection.

The cleaned dataset is then used to determine the pixel count for the middle row and the row distance. The final output includes extracted row data, row and plot positions, and plant pixel counts within each row section.

Supplementary Table 5: Description of Columns in 'data/Soybean_CanopyCover_Raw_data.csv'

Column Name	Description
Filename	The name of the processed image file including unique identifier for the plot.
Row_cluster_corr	The assigned cluster label for the detected middle row.
Lower_row_border	The lower boundary of the detected row (y-coordinate).
Upper_row_border	The upper boundary of the detected row (y-coordinate).
Sum_Pixel_row	The total number of pixels classified as green canopy cover in the row.
SD_Pixel_row	The standard deviation of the pixel count for every x-axis value within the row.
Row_middle_n	The median y-coordinate of the n-th detected row ($n = 1, \ldots, N$; where N is the total number of detected rows/clusters). If only one row/cluster is detected ($N = 1$), the plot width is defined by Plot_begin_Row_middle_n and Plot_end_Row_middle_n.
Plot_slope	The estimated slope of the row to correct for image distortion.
min_y_at_x0	The minimum y-coordinate at x = 0 (leftmost edge).
max_y_at_x0	The maximum y-coordinate at x = 0 (leftmost edge).
min_y_at_xmax	The minimum y-coordinate at the maximum x value (rightmost edge).
max_y_at_xmax	The maximum y-coordinate at the maximum x value (rightmost edge).
Sum_Pixel_plot	The total number of plant pixels detected in the entire plot.

Plot detection with canopy closure

When the canopy is closed, individual plots cannot be recognized easily on the presented images. The *get_plot_borders_mean()* function aggregates row-based position data across all measurements in early stages of the plants to determine plot boundaries. The input data is created with the above described *get_row_pixels* function. It cleans and removes outliers, computes upper and lower plot borders, and the average slope of the plot for later correction. This function helps localize the exact plot area in images, ensuring that extracted plant data is correctly positioned.

Then, the *get_green_canopy_plot_cover()* function processes segmented images to compute the green canopy cover for a full plot when the rows have completely covered the soil. It first loads the relevant image from each provided (*folder*) path, adjusts for plot borders and slope using previously estimated border values, and extracts plant pixels by filtering color channels. The total green fraction is calculated as the ratio of green pixels to total pixels. The

function then generates visualizations, saves processed images with segmentation overlays, and exports a .csv file containing pixel counts and canopy cover percentages.

Supplementary Table 6: Output variables when detecting plots with closed canopy

Variable	Description
Filename	The name of the processed image file.
x_r	The rounded x-coordinate used for segmentation.
Sum_Pixel_row	The total number of plant pixels detected in the plot.
SD_Pixel_row	Standard deviation of the pixel count within the plot.
Canopy_cover	The percentage of the image covered by green pixels.
Plot_width	The estimated width of the plot in pixels.
min_y_at_x0	Minimum y-coordinate at x = 0 (leftmost edge).
max_y_at_x0	Maximum y-coordinate at x = 0 (leftmost edge).
min_y_at_xmax	Minimum y-coordinate at the maximum x value (rightmost edge).
max_y_at_xmax	Maximum y-coordinate at the maximum x value (rightmost edge).

Stable extraction of canopy cover

First, a data set for canopy cover is created by the previously observed proportion of green pixels compared to the pixels in the entire plot. In parallel, Row_distance analyzes row spacing by detecting individual plant rows and calculating the distances between their middle points. To ensure accuracy, outliers are removed, and a rolling mean is applied to smooth variations in row spacing. Since some plots may have non-uniform plant distributions, it helps to adjust canopy cover estimates by normalizing green pixel counts based on row structure rather than just total pixels. The two datasets are then merged into one, which integrates both total canopy cover and row-based spacing measurements to calculate relative canopy cover. This ensures relative comparisons across genotypes and differences in planting density and row spacing. Outliers are removed before visualization, allowing for clearer growth trend analysis. This final dataset provides a standardized measure of canopy cover, making it more reliable for genotype comparisons and plant growth assessments over time (for the resulting csv file see: `data/Soybean_CanopyCover_data.csv`).

Detailed trait description

Supplementary Table 7: MIAPPE-compliant trait ontology for soybean phenotyping

Trait Label	Trait Name	SI Unit	Method ID	Method Name	Unit
CC	Canopy cover	percent	M01	High-resolution RGB image analysis (top canopy) using FIP platform with loess/spline-based smoothing	%
SPAD	Chlorophyll content (SPAD)	SPAD unit	M02	Handheld chlorophyll meter measurement (SPAD-502)	SPAD
TKW	Thousand kernel weight	g	M03	Automatic thousand seed weight determination using seed counter	g
Protein_content	Protein content	percent	M04	NIRS measurement with Perten 7200 Diode Array (three replicates in 2018 & 2022, single replicate otherwise)	%
Moisture_content	Moisture content	percent	M05	NIRS moisture content estimation (corrected using averaged 3-rep values where available)	%
Yield	Grain yield (11% moisture)	t/ha	M06	Weighed grain yield, corrected to a standardized moisture content of 11%	t/ha

Supplementary Figures

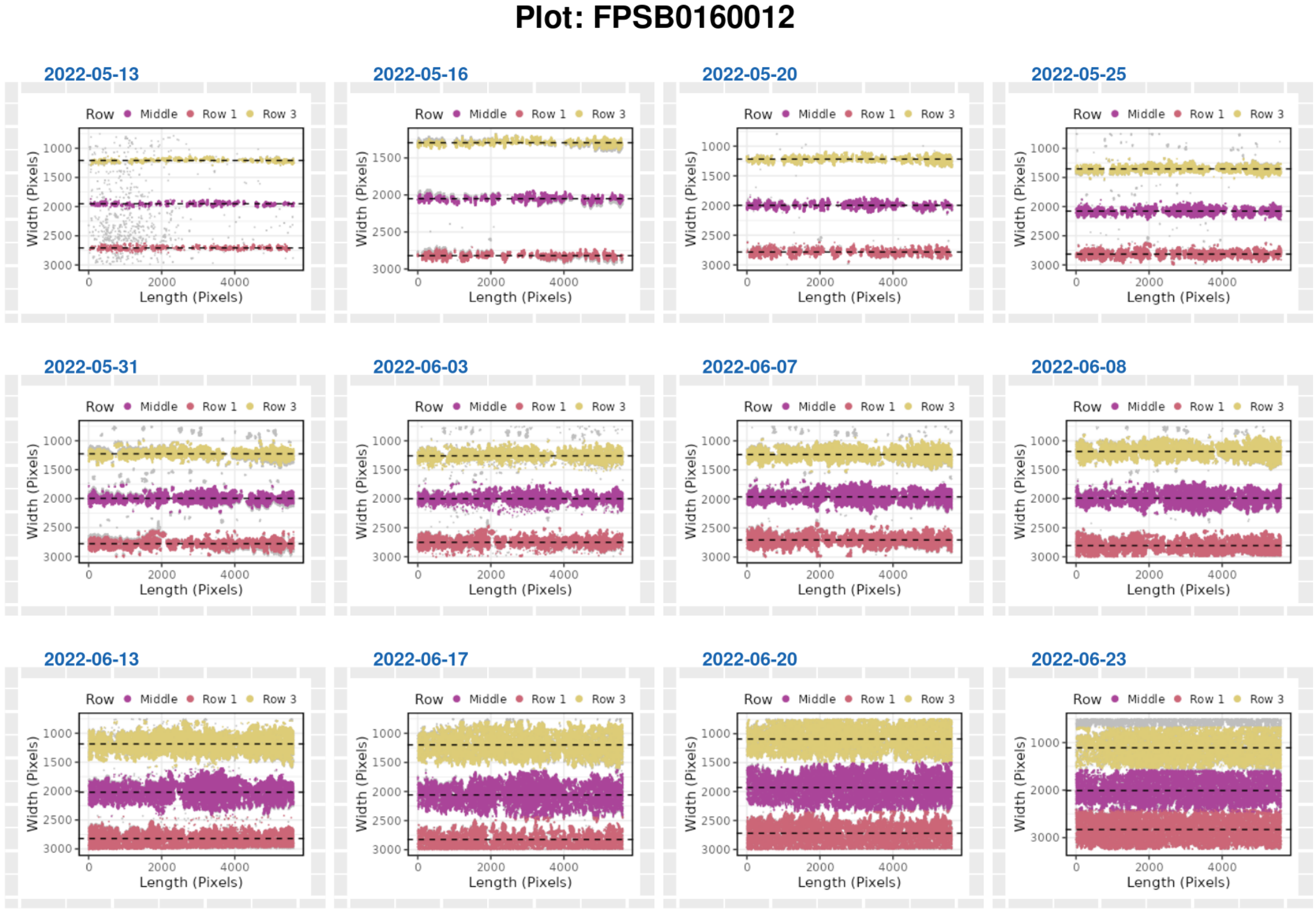

Supplementary Figure 1: Identification of the rows and middle row. Rows were detected to localize the plot and determine canopy cover (CC).

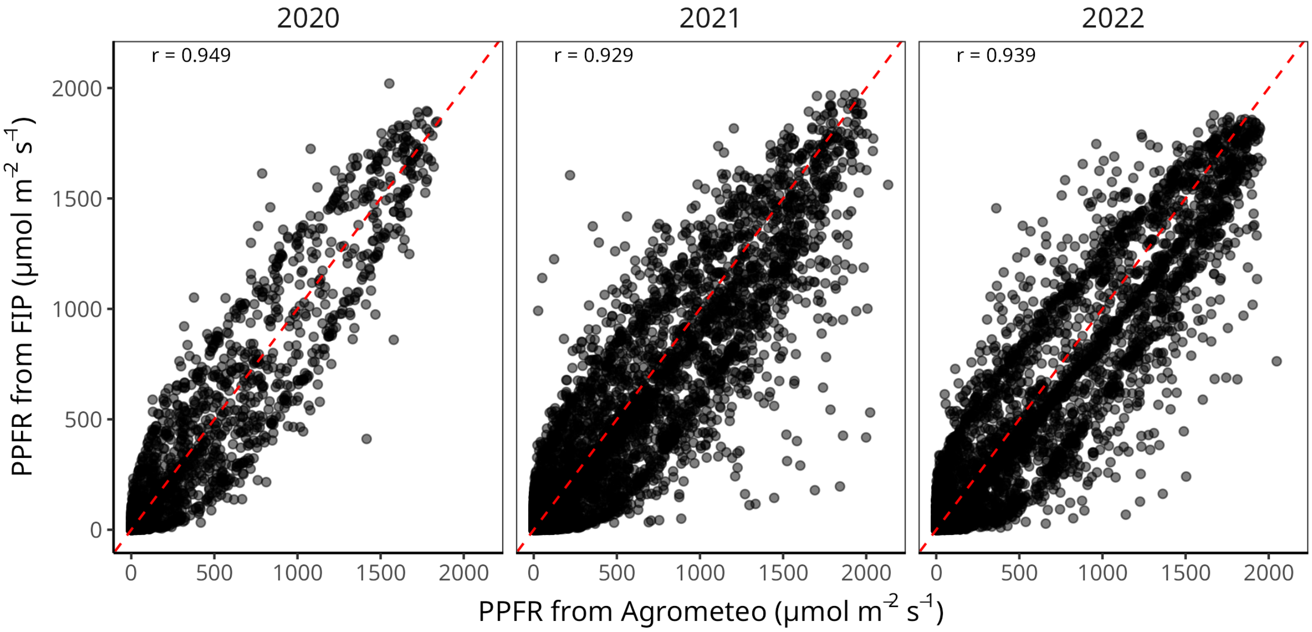

Supplementary Figure 2: Comparison of photosynthetic photon flux rate (PPFR) between the Agrometeo and the FIP weather station in Eschikon, Switzerland, for the overlapping years 2020–2022 at a one-hour resolution. Each panel shows data for one year with a dashed 1:1 reference line. Black labels indicate Pearson's correlation coefficients (r) between both sensors.

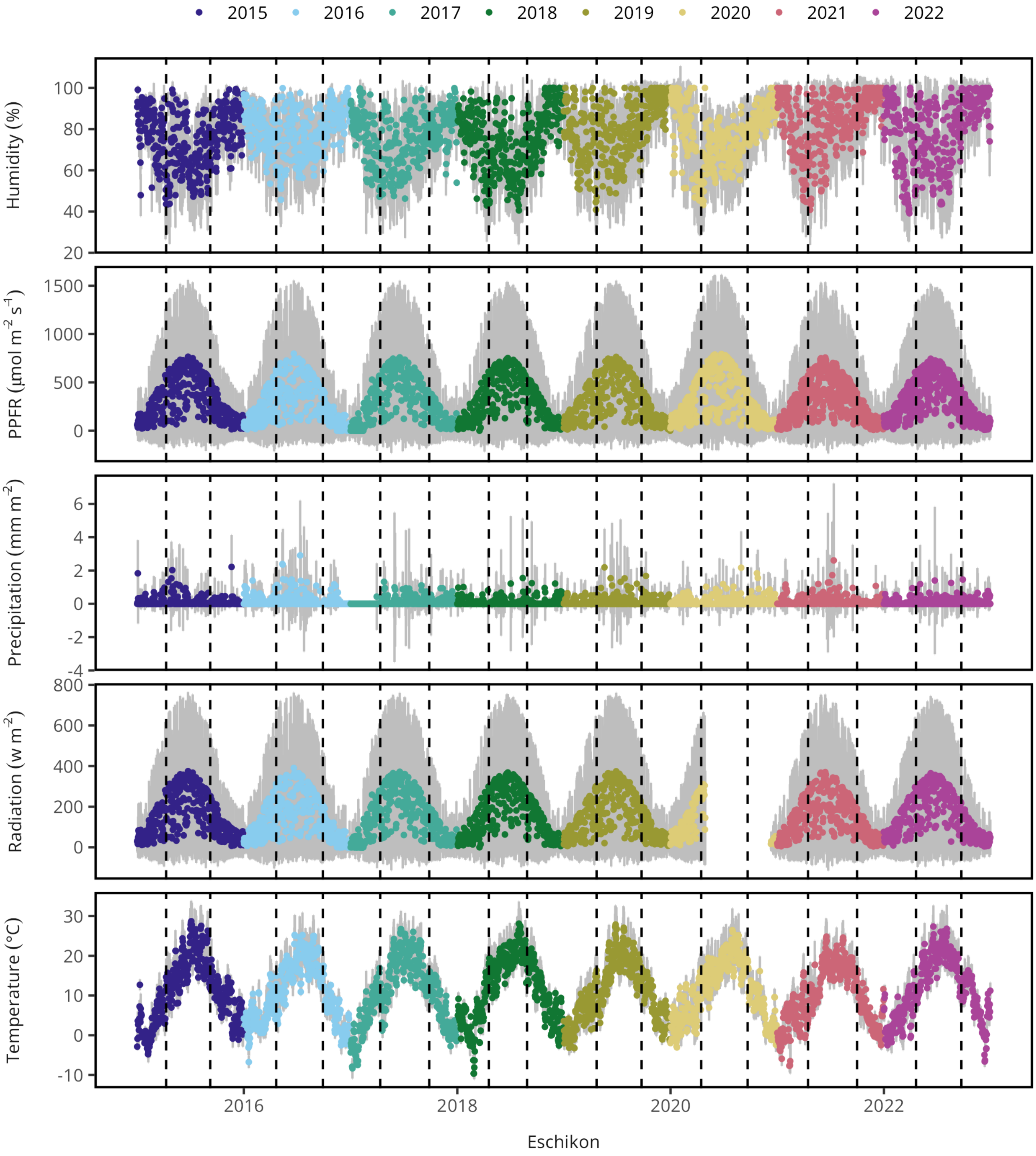

Supplementary Figure 3: Seasonal dynamics of weather variables recorded at Eschikon, Switzerland from 2015 to 2022. Daily means (points) and standard deviations (grey bars) are shown for temperature, photosynthetic photon flux density (PPFR), precipitation, relative humidity, and radiation. Vertical dotted lines indicate sowing respective harvest date.

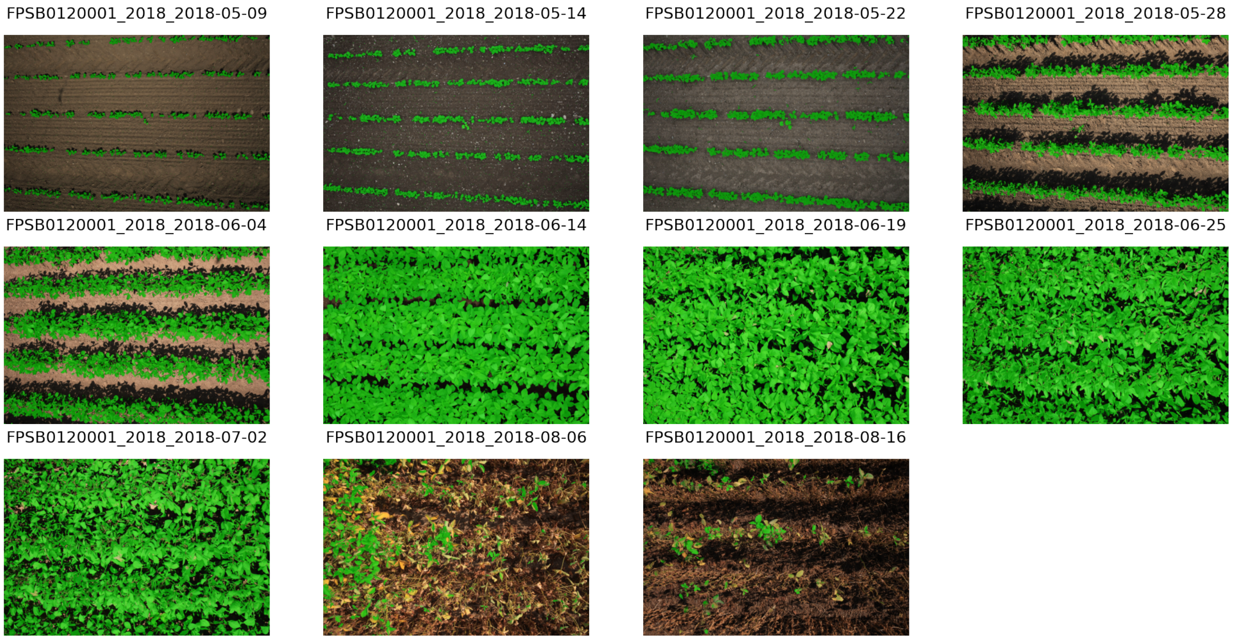

Supplementary Figure 4: Overlay of RGB images with their corresponding green-filtered segmentation masks for the 2018 growing season. Each image represents one observation date, showing a semi-transparent visualization of the extracted canopy regions (green areas) overlaid on the original RGB images. The figure illustrates the temporal progression of green canopy cover (CC) development for a representative plot in 2018.

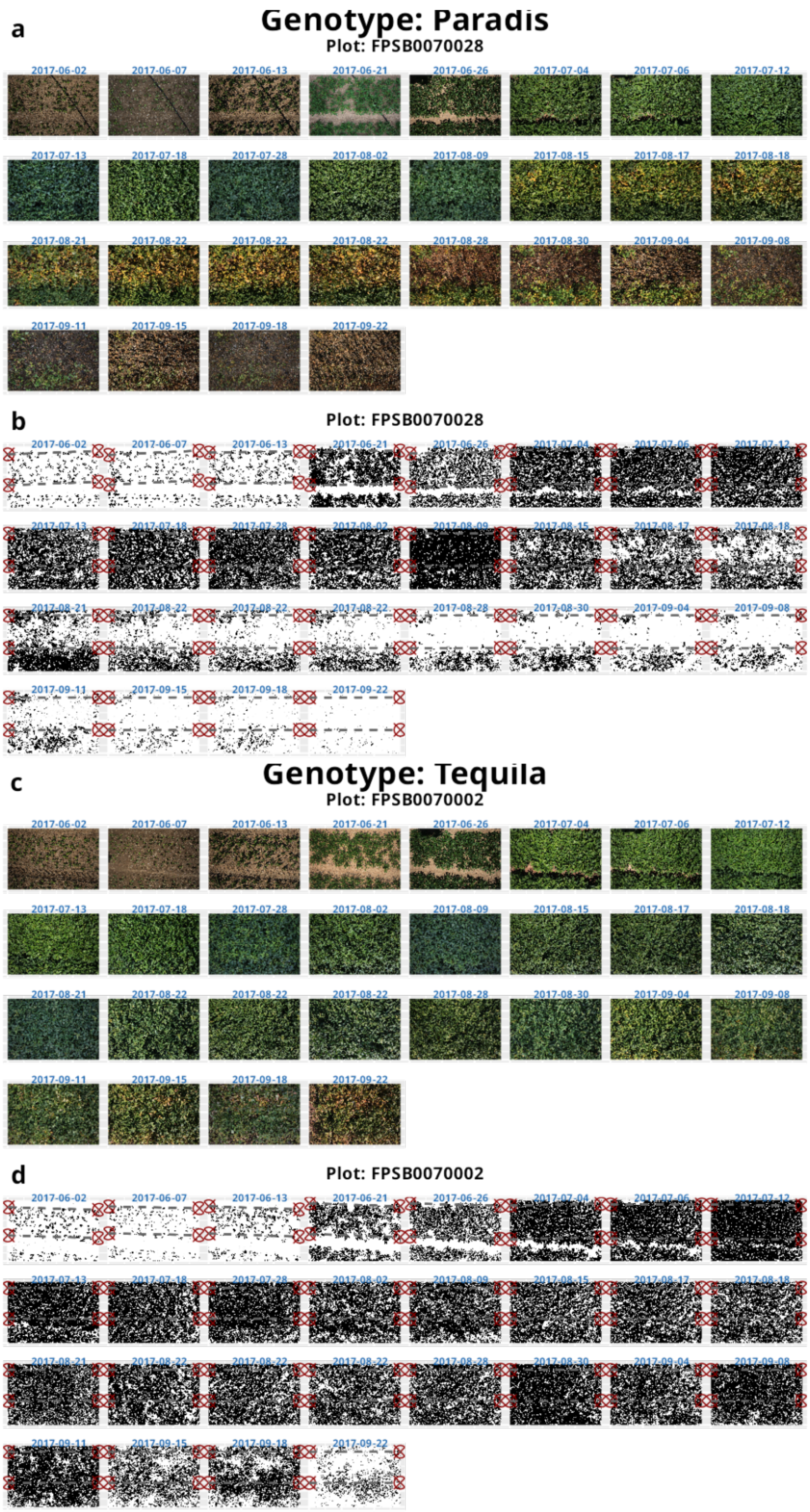

Supplementary Figure 5: Comparison of canopy development and segmentation masks for two soybean genotypes showing contrasting senescence timing in 2017 (trail FPSB007). a,b: Early senescence genotype 'Paradis' (maturity group 0000) showing faster canopy decline after peak cover. c,d: Late senescence genotype 'Tequila' (maturity group 00/0) maintaining green canopy longer into the season. Each row shows top-view RGB (a,c) and corresponding green-filtered segmentation masks across dates (b,d).

```python
from datasets import load_dataset
import pandas as pd
import matplotlib.pyplot as plt
import seaborn as sns

# --- Load dataset ---
ds = load_dataset("mikeboss/FIP1SOY", split="train")
print(ds)

# --- List available year/sites and filter (e.g. 7th = FPSB015) ---
unique_yearsites = ds.unique("yearsite_uid")
print("Available yearsite_uids:")
for i, y in enumerate(unique_yearsites):
    print(f"{i}: {y}")

example_yearsite = unique_yearsites[6]
print(f"\nSelected yearsite_uid: {example_yearsite}")
ds = ds.filter(lambda y: y == example_yearsite, input_columns="yearsite_uid")

# --- Convert to pandas and subset ---
df = ds.to_pandas()
sub = df.explode(["canopy_cover_values", "canopy_cover_dates"])
sub["canopy_cover_dates"] = pd.to_datetime(sub["canopy_cover_dates"])

n_genotypes = sub["genotype_id"].nunique()
print(f"\nNumber of unique genotypes in {example_yearsite}: {n_genotypes}")

# --- Plot ---
plt.figure(figsize=(9, 6))
sns.scatterplot(
    data=sub,
    x="canopy_cover_dates",
    y="canopy_cover_values",
    hue="genotype_id",
    style="replication",
    alpha=0.8,
    s=50,
)
plt.title(f"Canopy Cover over Time { {example_yearsite}")
plt.xlabel("Date")
plt.ylabel("Canopy Cover (%)")
plt.grid(True, alpha=0.3)
plt.tight_layout()
plt.legend(bbox_to_anchor=(1.05, 1), loc="upper left", title="Genotype / Rep")
plt.savefig("canopy_cover_timeseries.png", dpi=300, bbox_inches="tight")
plt.show()
```

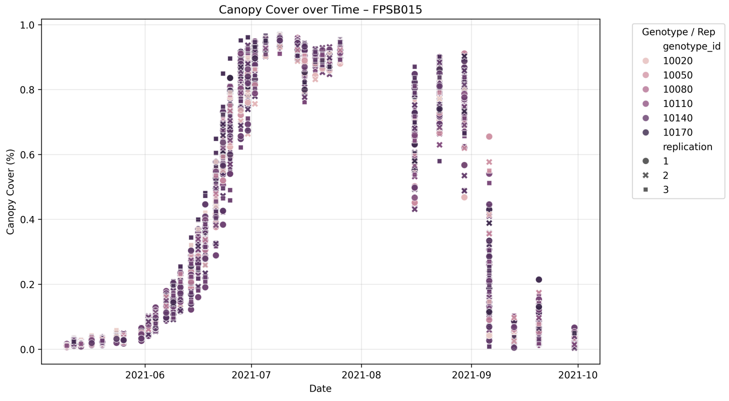

Supplementary Figure 6: Example Python code and resulting figure for visualizing canopy cover time series from the `mikeboss/FIP1SOY` dataset. Each point represents a single plot-level canopy cover observation over time, colored by genotype and shaped by replication.

References

[1] Hsien Ming Easlon **and** Arnold J. Bloom. "Easy Leaf Area: Automated digital image analysis for rapid and accurate measurement of leaf area". en. **in***Applications in Plant Sciences*: 2.7 (2014), **page** 1400033. ISSN: 2168-0450. DOI: `10.3732/apps.1400033`. URL: `https://onlinelibrary.wiley.com/doi/abs/10.3732/apps.1400033` (**urlseen** 29/11/2024).
